# Guideline adherence of tumor board recommendations in lung cancer and transfer into clinical practice

**DOI:** 10.1007/s00432-023-05025-1

**Published:** 2023-07-05

**Authors:** Julia Walter, Caroline Moeller, Blerina Resuli, Diego Kauffmann-Guerrero, Farkhad Manapov, Julien Dinkel, Jens Neumann, Julia Kovacs, Christian Schneider, Rudolf M. Huber, Amanda Tufman

**Affiliations:** 1 Department of Medicine V-Pneumology, University Hospital, Ludwig-Maximilians-University Hospital (LMU) Munich, Munich, Germany; 2 German Center for Lung Research (DZL), Giessen, Germany; 3 Department of Radiation Oncology, University Hospital, LMU Munich, Munich, Germany; 4 Department of Radiology, Asklepios Clinic Gauting, Gauting, Germany; 5 Institute of Pathology, Faculty of Medicine, LMU Munich, Munich, Germany; 6 Department of Thoracic Surgery, University Hospital, LMU Munich, Munich, Germany

**Keywords:** Multidisciplinary tumor board, Thoracic malignancies, Guidelines

## Abstract

**Purpose:**

Evaluating patients and treatment decisions in a multidisciplinary tumor board has led to better quality of care and longer survival in cancer patients. The aim of this study was to evaluate tumor board recommendations for thoracic oncology patients regarding guideline adherence and transferal of recommendations into clinical practice.

**Methods:**

We evaluated tumor board recommendations of the thoracic oncology tumor board at Ludwig-Maximilians University (LMU) Hospital Munich between 2014 and 2016. We compared patient characteristics between guideline-adherent and non-guideline-adherent recommendations, as well as between transferred and non-transferred recommendations. We used multivariate logistic regression models to evaluate factors associated with guideline adherence.

**Results:**

Over 90% of recommendations by the tumor board were either adherent to the guidelines (75.5%) or over fulfilling guidelines (15.6%). Almost 90% of recommendations were transferred to clinical practice. If a recommendation was not according to the guidelines, the reason was mostly associated with the general condition (age, Charlson comorbidity index, ECOG) of the patient or due to the patients’ request. Surprisingly, sex also had a significant influence on the guideline adherence of recommendations, with females being more likely to get recommendations not according to the guidelines.

**Conclusion:**

In conclusion, the results of this study are promising, as the guideline adherence of recommendations as well as the transferal of recommendations into clinical practice were high. In the future, a special focus should be put on fragile patients as well as female patients.

**Supplementary Information:**

The online version contains supplementary material available at 10.1007/s00432-023-05025-1.

## Background

The treatment of patients with lung cancer can be quite complex as the decision for the best therapy depends not only on the size and location of the tumor but also on molecular and immunological tumor characteristics, as well as individual patient characteristics. In addition, with the introduction of targeted agents and immunotherapy, a further layer of complexity was added. Cooperation and exchange of knowledge between different disciplines from pathology over surgery, to radiology and pneumology can help finding the best treatment path. In order to put an emphasis on multidisciplinary cooperation, on behalf of the German Cancer Society (DKG) (Bundesministerium für Gesundheit 2012) OnkoZert (2023), an independent institution, can certify hospitals in Germany as specialized lung cancer centers. The aim of certifying centers is establishing holistic patient-oriented medical treatment that follows national guidelines from prevention and early diagnosis, to diagnostic approach, therapy, and follow-up care (Bundesministerium für Gesundheit 2012). One of the requirements to be certified is the introduction of weekly multidisciplinary tumor board meetings (Bundesministerium für Gesundheit 2012). Participants of the tumor board meetings are thoracic surgeons, pneumologists, a thoracic oncologist, pathologists, radiologists, and radiation oncologists. Tumor conferences include the detailed evaluation of tumor imaging, pathology reports, and the clinical situation of the patients. The result is a consented therapy recommendation based on national and international guidelines, current research, and the clinical experience of each specialist, adapted to each individual patient. Often, tumor board meetings also discuss cases of patients presented by external physicians and have therefore become part of the general care in oncological patients (Devitt et al. 2010). Studies have shown that these types of multidisciplinary recommendations have improved treatment quality compared to individual case-by-case recommendations (Specchia et al. 2020; Bydder et al. 2009; Lamb et al. 2013).

Some studies have evaluated guideline adherence of tumor board recommendations. A study from the Netherlands investigating factors influencing multidisciplinary tumor board recommendations in stage III non-small cell lung cancer found that age and performance status were the most important predictors of not recommending guideline recommended therapy (Ronden et al. 2021). Another study from the US evaluated the effectiveness of molecular tumor board recommendations in a small group of patients and found that adherence to tumor board recommendations resulted in a high response rate to treatment and good progression-free survival (Koopman et al. 2020). A preliminary analysis of tumor board recommendations in Northern Germany found that patients with a complete adherence to the tumor board recommendations had a significantly longer median survival compared to patients with non-adherence or partial adherence (Roeper et al. 2021). However, as of yet, there is no study combining the evaluation of guideline adherence of recommendations and transfer of recommendation into clinical practice in thoracic cancers of all stages.

Therefore, the aim of this study was to first evaluate the guideline adherence of recommendations by our thoracic multidisciplinary tumor board and assess reasons for non-adherence as well as factors influencing non-adherence. Second, we aimed to evaluate the transfer of tumor board recommendations into clinical practice, and again analyze reasons and factors impacting non-adherence.

## Methods

### Study design and study population

In this retrospective study, we evaluated recommendations of thoracic tumor board meetings at LMU Hospital Munich between January 2014 and January 2016. At the LMU Hospital Munich, the thoracic multidisciplinary tumor board takes place on a weekly basis. We included all patients presented to the tumor board meetings with a diagnosis of a thoracic malignancy. We excluded patients with a mediastinal or pleural malignancy, carcinoid tumors, or lung metastases. We also excluded patients with a suspected thoracic malignancy without histological confirmation, and patients with a cancer of unknown primary (CUP). Furthermore, we excluded patients with third-line or more than third-line therapy who had been previously presented for first- or second-line therapy before the start of the inclusion period.

We collected information on age, sex, tumor histology, tumor stage, performance status measured in Eastern Cooperative Oncology Group (ECOG) score, and comorbidity. Comorbidity was evaluated using Charlson comorbidity index (CCI) (Charlson et al. 1987), as well as CCI grade. Information on tumor stage, ECOG and comorbidities was missing in a few patients. For stage, ECOG and CCI grade we created an extra category called ‘unknown’. Mean values for CCI index were calculated only for patients without missing information on comorbidity. All data were accumulated during the tumor board meetings, from official tumor board protocols, and from electronic patient records.

### Ethic approval

The ethics committee of the Ludwig-Maximilians University Munich (reference number 475-16 UE) approved this non-interventional study. The study was conducted in accordance with the Declaration of Helsinki, Good Clinical Practice guidelines, and local ethical and legal requirements.

### Evaluation of tumor board meetings

We collected the following information for each patient regarding tumor board presentations: number of presentations, recommendation, adherence to guideline, reasons for non-adherence, transfer of recommendation to clinical practice, and reasons for non-transfer.

**Fig. 1:**
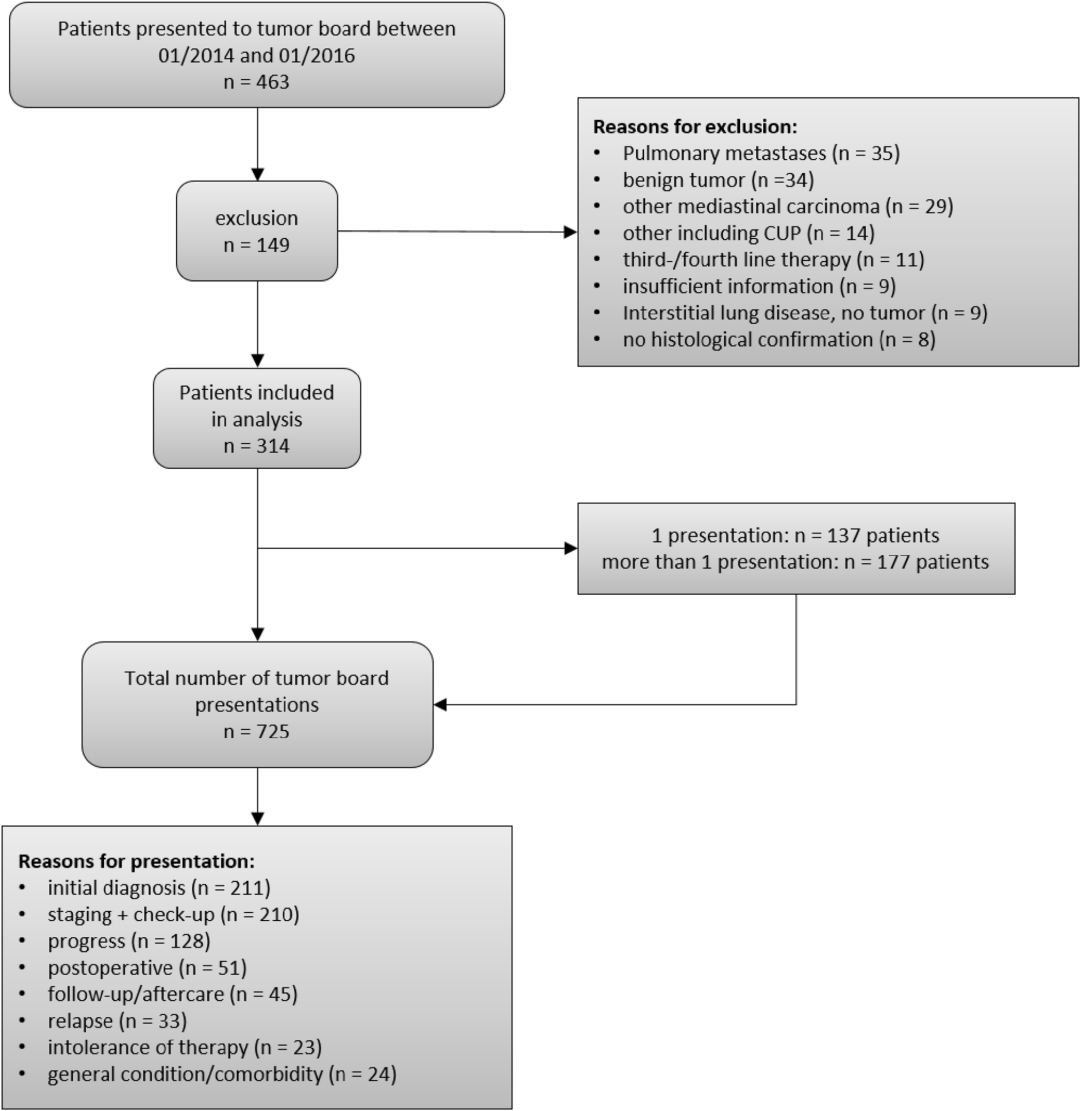
Patient flow diagram. *CUP* cancer of unknown primary

Guideline adherence was evaluated using the Standard operating procedures (SOP) of the thoracic oncology center Munich (Belka 2014). The SOPs of the thoracic oncology center Munich are based on the German S3-Guideline (Goeckenjan et al. 2010) and the recommendations of the manual on tumors of the lung and the mediastinum of the tumor center Munich (Huber 2014). Figures 1 to 5 of the supplemental material include a detailed description of therapies in NSCLC and SCLC by stage recommended in the SOPs. Hereafter, we refer to the SOPs as guideline. Tumor board recommendations were categorized into according to guideline, extended treatment, and limited treatment. Extended treatment referred to treatment recommendations beyond what the guidelines include, whereas limited treatment referred to recommendations that were less than what was included in the guidelines. An example for extended treatment would be therapy with more recent tyrosine kinase inhibitors (TKIs) as some were not yet included in the SOPs during the study period, but approved for therapy in Germany. Reasons for recommendations not according to guidelines were categorized into due to general condition/comorbidities/therapy intolerance, not (yet) included in guidelines/inclusion in clinical trial, justified individual decision, patient request, and other reason.

Evaluation of transfer of tumor board recommendations to clinical practice and adherence to guidelines was performed in a 2 step process. First, we assessed whether the recommendation made by the tumor board was delivered to the patient. Successful transfer of a recommendation was defined as transfer of the recommended treatment within 4 weeks or until the next presentation to the tumor board. In a second step, we categorized successful transfer according to guideline adherence of the tumor board recommendation from the previous analysis. Transfer then was categorized into no transfer (e.g., no therapy, therapy not as recommended), transfer according to guideline (transfer of guideline-adherent recommendation), and transfer not according to guideline (transfer of non-guideline-adherent recommendation). Reasons for non-transferal of recommendations into clinical practice were categorized into due to patient death, general condition/comorbidities, patient request, external therapy, no further information (e.g., due to treatment in a different hospital), and other (e.g., change of tumor stage or mutation status, imprecise documentation).

### Statistical analysis

We evaluated guideline adherence and transfer into clinical practice for all recommendations by the tumor board. However, for comparisons of characteristics, we only included the first recommendation for each patient.

We presented results for categorical variables as absolute and relative frequencies and compared them between groups using Chi^2^-test or Fisher-exact test. Numerical variables were reported using means and standard deviations and group comparison was made using analysis of variance (ANOVA). We used multivariate logistic regression models to evaluate the association of age, sex, histology, stage, ECOG, and CCI with guideline adherence of recommendations. For the exploratory analysis of factors influencing guideline adherence, we used a logistic regression model. Here, guideline adherence was defined as according to guideline and extended recommendations, compared to limited recommendations. In addition, we only used patients with complete information on all covariates for the regression model. Statistical significance was determined using two-sided *p* values.

Data analysis was performed using R Version 4.0.0 and RStudio Version 1.4. Tables and figures were created in Microsoft Excel and PowerPoint.

## Results

In total, 463 patients were presented to the thoracic tumor board between January 2014 and January 2016. Of these, 149 patients were excluded due to the inclusion and exclusion criteria leaving 314 patients for this analysis (see Fig. 1 for details). The mean age of patients was 67.4 years (sd = 10.4), 55.1% (*n* = 173) were male and 44.9% (*n* = 141) were female.

The distribution of histologic subtypes was 59.9% (*n* = 188) adenocarcinoma, 22.6% (*n* = 71) squamous-cell carcinoma (SCC), 12.1% (*n* = 38) small cell carcinoma (SCLC) and 5.4% (*n* = 14) other histologic subtypes.

The majority of patients had stage IV disease (55.7%, *n* = 175), 21.7% had stage III (*n* = 68), 9.2% had stage II (*n* = 29), 12.4% had stage I (*n* = 39), and for 3 patients, stage was unknown (1.0%). Mean CCI was 1.12 (sd = 1.26), and 29.3% (*n* = 92) had ECOG 0, 37.2% (*n* = 117) ECOG 1, 14.6% (*n* = 46) ECOG 2, 9.6% (*n* = 30) ECOG 3 or more, and for 9.2% (*n* = 29), ECOG was unknown.

### Evaluation of guideline adherence of recommendations

Of the 314 patients, 75.5% (*n* = 237) received recommendations according to the guidelines in their first presentation to the tumor board. For 15.6% (*n* = 49) of patients, the recommendation was for extended treatment, and for 8.9% (*n* = 28) of patients, the recommendations were limited. Recommendations for extended treatment were therapies not yet included in the guidelines, as well as inclusion of patients in clinical trials (63.6%, *n* = 31), justified individual decision (28.6%, *n* = 14), general condition/comorbidity/therapy intolerance (4.0%, *n* = 2), patient request (2.0%, *n* = 1), and other reasons (2.0%, *n* = 1). An example for a justified individual decision would be the case of a 55-year-old patient presented with stage IV adenocarcinoma with brain metastases, in good general condition, who received brain radiation, followed by chemotherapy and tumor resection. Reasons for recommendations of limited treatment were general condition/comorbidity/therapy intolerance (78.6%, *n* = 22), patient request (10.7%, *n* = 3), and other (10.7%, *n* = 3). Table 1 shows baseline patient characteristics and causes of non-adherence to guidelines including general condition/comorbidity/therapy intolerance, not (yet) included in guidelines/inclusion in clinical trial, and justified individual decision.

**Table 1:** Patient characteristics stratified by guideline adherence of recommendation

	Recommendation according to guidelines (*n* = 237)		Recommendation for extended treatment (*n* = 49)		Recommendation for limited treatment (*n* = 28)		*p* value
	Mean	sd	Mean	sd	Mean	sd	
Age in years	67.8	9.75	63	12.1	71.7	9.84	0.001
CCI^a^	1.05	1.12	0.98	1.3	1.96	1.91	0.001

	*n*	%	*n*	%	*n*	%	*p* value
Sex							
Male	131	55.3%	31	63.3%	11	39.3%	0.13
Female	106	44.7%	18	36.7%	17	60.7%	
Histology							
Adenocarcinoma	147	62.0%	31	63.3%	10	35.7%	0.01
Squamous-cell carcinoma	52	21.9%	12	24.5%	7	25.0%	
SCLC	30	12.7%	3	6.1%	5	17.9%	
Other	8	3.4%	3	6.1%	6	21.4%	
Stage							
I	34	14.4%	2	4.1%	3	10.7%	0.002
II	21	8.9%	1	2.0%	7	25.0%	
III	56	23.6%	7	14.3%	5	17.9%	
IV	124	52.3%	39	79.6%	12	42.9%	
Unknown	2	0.8%	0	0.0%	1	3.6%	
ECOG							
0	78	32.9%	11	22.5%	3	10.7%	0.005
1	90	38.0%	17	34.7%	10	35.7%	
2	25	10.6%	12	24.5%	9	32.1%	
3 or higher	20	8.4%	6	12.2%	4	14.3%	
Unknown	24	10.1%	3	6.1%	2	7.1%	
CCI grade							
1	89	37.6%	23	46.9%	6	21.4%	0.04
2	110	46.4%	15	30.6%	16	57.1%	
3	25	10.6%	5	10.2%	4	14.3%	
4	2	0.8%	1	2.0%	2	7.1%	
Unknown	11	4.6%	5	10.2%	0	0.0%	

Characteristics of patients presented to the tumor board between January of 2014 and January 2016, stratified by guideline adherence of recommendation made by the tumor board. Numerical variables are presented as means with standard deviation, and categorical variables as absolute and relative frequencies. *p* values are from ANOVA and Chi^2^-test or Fisher-exact test, for numerical and categorical variables, respectively

*sd* standard deviation, *CCI* Charlson Comorbidity Index, *SCLC* small cell lung cancer, *ECOG* Eastern Cooperative Oncology Group

^a^Calculated for patients without missing information regarding comorbidities

We found that the mean age of patients receiving a guideline-adherent recommendation (67.8 years), or a recommendation for extended treatment (63.0 years) was significantly lower (*p* value = 0.001), compared to patients with a recommendation for limited treatment (71.7 years). The distribution of histological subtype also differed significantly between the groups (*p* value = 0.02). In the group of patients with recommendations for limited treatment, there was a lower proportion of adenocarcinoma (35.7% vs. 63.3% vs. 62.0%), a larger proportion of SCLC (17.9% vs. 6.1% vs. 12.7%), and a larger proportion of other less common tumors (21.4% vs. 6.1% vs. 3.4%) compared to patients with extended and guideline-adherent recommendations. ECOG score differed significantly between the groups (*p* value = 0.002), with the largest proportion of patients with ECOG ≥ 3 in patients with limited treatment recommendations. Furthermore, mean CCI was 1.96 in these patients which was significantly (*p* value = 0.04) higher than in patients with recommendations according to the guidelines (mean = 1.05) and in patients with extended treatment recommendations (mean = 0.98). For exact comparison of characteristics between the three groups, please refer to Table 1.

Multivariate logistic regression model of guideline adherence (recommendation according to guideline + extended treatment, vs. limited treatment) confirmed the significant influence of comorbidity (OR = 0.69, *p* value = 0.03) and partially confirmed the significant influence of stage (stage II vs. stage IV: OR = 0.17, *p* value = 0.01). In addition, it added the significant influence of sex, with an odds ratio of 3.0 in favor of males (*p* value = 0.02). Table 2 displays results of the logistic regression model.

**Table 2:** Results of logistic regression analysis of guideline adherence of recommendations

	OR	*β*	se	*z* value	*p* value
Age in years	0.96	− 0.04	0.03	− 1.35	0.18
Male vs. female	3.02	1.11	0.49	2.24	0.02
ECOG 1 vs. 0	0.56	− 0.58	0.72	− 0.80	0.42
ECOG 2 vs. 0	0.25	− 1.39	0.80	− 1.74	0.08
ECOG 3 or higher vs. 0	0.46	− 0.77	0.87	− 0.88	0.38
Other histology vs. adenocarcinoma	0.22	− 1.51	0.82	− 1.84	0.07
Squamous-cell carcinoma vs. adenocarcinoma	0.79	− 0.23	0.58	− 0.40	0.69
SCLC vs. adenocarcinoma	0.40	− 0.91	0.69	− 1.33	0.18
Stage II vs. I	0.17	− 1.78	0.93	− 1.92	0.05
Stage III vs. I	0.76	− 0.27	0.93	− 0.29	0.77
Stage IV vs. I	0.97	− 0.03	0.85	− 0.03	0.98
CCI	0.69	− 0.37	0.17	− 2.21	0.03

Odds ratios with *p* values from logistic regression model of guideline adherence (guideline + more than guideline) vs. non-guideline adherence of recommendations made by the tumor board. Analysis of patients with complete information on all variables (*n* = 278)

*OR* odds ratio, *ECOG* Eastern Cooperative Oncology Group, *SCLC* small cell lung cancer, *CCI* Charlson Comorbidity Index

### Transfer of recommendations into clinical practice

In total, the 314 patients were presented 725 times to the tumor board and all received a therapy recommendation. Of the 314 patients, 137 were presented once, and 177 patients were presented to the tumor board more than once. Of all recommendations, 81.0% (*n* = 587) were transferred into clinical practice. Of these, 89.6% (*n* = 526) were guideline adherent. 138 recommendations of 123 patients were not transferred, the reason for this was death of the patient in 6.5% (*n* = 9), general condition/comorbidities in 8.0% (*n* = 11), patient request in 21.7% (*n* = 30), and other reasons 5.8% (e.g., imprecise documentation, change of stage or mutation status). In 58.0% of non-transferred recommendations, no further information was available due to loss to follow-up, as patients had been seeking a second opinion, or were treated elsewhere. Characteristics of patients with loss to follow-up can be examined in Table 2.

After the first presentation to the tumor board, recommendations were transferred to clinical practice in 77.1% of cases (*n* = 242), and of these, 222 were according to the guidelines and 20 not according to guidelines. Only ECOG status was significantly associated with the transfer of recommendations to clinical practice (*p* value 0.02); however, there was no consistent trend. A comparison of all characteristics can be found in Table 3.

**Table 3:** Patient characteristics stratified by transfer of recommendation into clinical practice

	No transfer of recommendation (*n* = 72)		Transfer according to guideline (*n* = 222)		Transfer not according to guideline (*n* = 20)		*p* value
	Mean	sd	Mean	sd	Mean	sd	
Age in years	68.0	11.2	67.2	9.95	65.2	12.8	0.32
CCI	1.27	1.27	1.02	1.13	2.16	1.95	0.32

	*n*	%	*n*	%	*n*	%	*p* value
Sex							
Male	41	56.9%	121	54.5%	9	45.0%	0.64
Female	31	43.1%	101	45.5%	11	55.0%	
Histology							
Adenocarcinoma	43	59.7%	134	60.4%	9	45.0%	0.08
Squamous-cell carcinoma	11	15.3%	55	24.8%	6	30.0%	
SCLC	12	16.7%	25	11.3%	2	10.0%	
Other	6	8.3%	8	3.6%	3	15.0%	
Stage							
I	3	4.2%	33	14.9%	3	15.0%	0.14
II	8	11.1%	19	8.6%	1	5.0%	
III	19	26.4%	47	21.2%	2	10.0%	
IV	42	58.3%	120	54.1%	14	70.0%	
Unknown	0	0.0%	3	1.4%	0	0.0%	
ECOG							
0	16	22.2%	73	32.9%	3	15.0%	0.01
1	29	40.3%	83	37.4%	5	25.0%	
2	7	9.7%	30	13.5%	9	45.0%	
3 or higher	9	12.5%	19	8.6%	2	10.0%	
Unknown	11	15.3%	17	7.7%	1	5.0%	
CCI grade							
1	23	31.9%	102	45.9%	5	25.0%	0.08
2	33	45.8%	89	40.1%	9	45.0%	
3	7	9.7%	19	8.6%	3	15.0%	
4	3	4.2%	4	1.8%	2	10.0%	
Unknown	6	8.3%	8	3.6%	1	5.0%	

Characteristics of patients presented to the tumor board between January of 2014 and January 2016, stratified by transfer of recommendation into clinical practice and guideline adherence of transfer. Numerical variables are presented as means with standard deviation, and categorical variables as absolute and relative frequencies. *p* values are from ANOVA and Chi^2^-test or Fisher-exact test, for numerical and categorical variables respectively

*sd* standard deviation, *CCI* Charlson Comorbidity Index, *SCLC* small cell lung cancer, *ECOG* = Eastern Cooperative Oncology Group

## Discussion

Overall, we found that over 90% of recommendations by the thoracic tumor board at the LMU Hospital Munich were either adherent to the corresponding guidelines (75.5%) or recommendations for extended treatment (15.6%). Of all recommendations included in the analysis, almost 90% were transferred to clinical practice. If a recommendation was not according to the guidelines, the reason was mostly associated with the general condition (age, CCI, ECOG) of the patient or due to the patients’ request. Surprisingly, sex also had a significant influence on the guideline adherence of recommendations, with females being more likely to get recommendations for limited treatment.

Compared to other studies assessing tumor board recommendations and guideline adherence in treatments, the proportion of guideline adherence of recommendations by our tumor board as well as the transfer of recommendations into clinical practice was high. An analysis of multidisciplinary tumor board recommendations in stage III NSCLC found that the preferred approach of concurrent chemoradiotherapy and/or surgery was recommended in 61% of patients, with only 48% of patients actually receiving it (Ronden et al. 2021). A different study of recommendations by a molecular tumor board reported an adherence to recommendations in 81% of cases (Koopman et al. 2020). Preliminary results of a study of tumor board recommendations in Northern Germany showed that 78% of recommendations by the tumor board were completely adhered to Roeper et al. (2021).

Age, comorbidity level, and ECOG score were significantly associated with recommendations adherent to guidelines in the univariate analysis. CCI was also significantly associated with recommendation adherence in the multivariate analysis. Patients with recommendations extended treatment were younger, had lower comorbidity scores and were more likely to have an ECOG lower than 2. Patients with recommendations for limited treatment were older, had higher comorbidity scores and were more likely to have an ECOG of 3 or higher. It is unsurprising that there is often consensus about offering extended therapies to young adults patients, with good baseline performance status and otherwise long life expectancy. Due to their age and general condition, they are more likely to benefit and tolerate these therapies. We found that increasing age was significantly associated with underuse of guideline recommended chemotherapy in a study from 2012 (Salloum et al. 2012). Generally, patients with comorbidities are excluded from randomized controlled trials and so guideline recommendations for patients with comorbidities are usually based on weak to moderate evidence or are not available at all.

In addition, in a study reviewing multidisciplinary tumor board recommendations in stage III NSCLC, age over 70 and ECOG score ≥ 2 were the most important predictors of not recommending the radical intent treatment (Ronden et al. 2021).

In our analysis, 12% of all female patients, received recommendations for limited treatment, whereas only 6% of male patients received a recommendation for limited treatment. This finding is surprising, even though for almost 50% of these women the reason for the recommendation was due the general condition of the patient. A difference in guideline adherence according to sex was not reported in previous studies.

However, the difference of guideline adherence regarding sex might be in part explained by the fact that clinical trials tend to include fewer females leading to less evidence about safety and efficacy in females. Historically, women have been underrepresented in clinical trials, but despite efforts to improve this gap, recent studies suggest that they are still common (Steinberg et al. 2021; Sosinsky et al. 2022). Compared to men women are more likely to be diagnosed at a younger age and with adenocarcinoma, they often have a family history of cancer, and a lack of smoking history. Often a tumor with a driver mutation is found. Current screening guidelines select participants mostly regarding age and smoking history and therefore may underestimate risk in women. In the NELSON trial (Ru Zhao et al. 2011; de Koning et al. 2020), a randomized controlled study that evaluated the role of low-density computed tomography (LDCT) in heavy smokers, women comprised only 16% of participants and were not included in the main outcome analysis. The lack of guidelines and risk assessment for light or never smokers predispose women with lung cancer to be missed with current screening recommendations.

Histology was also significantly associated with deviation from the guidelines in our univariate analysis. The proportion of patients with adenocarcinoma was higher in the in the group of patients with guideline adherence, and SCLC was more frequent in the group of patients with recommendations for limited treatment. This reflects in part major progress made in the last years in the field of NSCLC, primarily in adenocarcinoma and SCC, compared to SCLC. Moreover, adenocarcinoma patients harbor mutations in oncogenic drive genes, allowing for a molecularly stratified, personalized treatment with approved target therapies. The last decade has seen the emergence of various targeted therapies for treatment in adenocarcinoma, and immune therapies in SCC, whereas few improvements have been made in the treatment of SCLC in the past few decades, where most advances have been restricted to immunotherapy and improved radiation approaches.

Almost 80% of patients with recommendations for extended treatment were diagnosed with stage IV disease. This might reflect the multimodal treatment of young and fit patients with oligometastatic disease. In the group of patients with recommendations for limited treatment, not all presented with advanced disease at diagnosis but probably age and comorbidities influenced treatment decisions.

This interpretation is strengthened by stage not being consistently significant in the regression analysis, when adjusted by age and comorbidity.

One aspect that needs to be considered when analyzing guideline adherence is that clinicians are increasingly dependent on guidelines to keep up with the latest developments in oncology and guidelines are followed to avoid malpractice suits. A key challenge in this area is the rapid change with respect to new targets and technologies, and recommendations need to accommodate new emerging data. The process for guideline development takes a long time and when the guidelines are published they might lack information regarding the latest updates, e.g., from international conferences. The vast majority of medical oncologist in their daily practice refer to the National Comprehensive Cancer Network (NCCN) Guidelines. All active NCCN Guidelines are reviewed and updated at least annually, but this might differ for national Guidelines of a country. For example in Germany, the last updated guidelines for lung cancer were published in December 2022. The previous version was published in February 2018. Between 2018 and 2022, we have witnessed a revolution in the treatment of NSCLC with significant advances in early detection and therapeutic modalities. In Germany, not all clinicians refer to the international Guidelines in their daily practice, and for this reason, the role of tumor boards is crucial in patients cancer care. The task of the tumor boards is to formulate therapy recommendations that are based on scientific evidence on the one hand and individually adapted to each patient on the other hand.

The patient population in our study largely reflected the general population of thoracic oncology patients in Germany, only the distribution of sex and histological type differed to some extent. Mean age at diagnosis in Germany is 69 years for women and 70 years for men (Robert Koch-Institut 2019); in our study, mean age was 67.4 years. Our study included 45% female patients and 55% male patients, whereas according to the federal Robert Koch Institute (RKI), the proportion of female patients between 2014 and 2016 was around 37–38% (Robert Koch-Institut 2019). This difference was also reflected by an overrepresentation of adenocarcinoma in our study compared to the whole of Germany (59.9% vs. 43%) and underrepresentation of SCLC (12.2% vs. 20%) (Robert Koch-Institut 2019). The discrepancy in histology distribution in our analysis can be explained by the fact that females present more often with adenocarcinoma rather than SCLC, which is more common in male patients. Stage distribution was similar to the one reported by RKI in 2015 and 2016 (Robert Koch-Institut 2019).

One limitation of this study was the completeness of information on patient characteristics and follow-up. We did not have information on ECOG score for 29 (9.2%) patients. In addition, we did not have information on whether recommendations of the tumor board were transferred into clinical practice for patients who came for a second opinion or patients treated outside LMU Hospital Munich. This accounted for 80 recommendations, and 58% of recommendations categorized as not transferred. In addition, this analysis evaluated tumor board recommendations of one tumor board in a single lung tumor center in Germany, which does not necessarily reflect the whole population of lung cancer patients. In addition, this study was explorative in nature, and therefore, our results cannot be used in a way that results from confirmatory studies can be. However, they can be used for hypothesis generation in this field. Further, the categorization of recommendations according and not according to guidelines are dependent on the selection of guidelines to follow. Especially, regarding stage III cancer, guidelines were non-homogenous, therefore, categorization was not always easy.

A strength of this analysis is the number of patients included and the number of recommendations evaluated, and inclusion of all histological subtypes and stages. Another strength is the detailed documentation of reasons for non-guideline adherence and non-transferal of recommendations. Therefore, this study gives a comprehensive overview of the work of our multidisciplinary tumor board.

## Conclusions

Multidisciplinary care is the cornerstone of lung cancer treatment. It facilitates the delivery of high-quality lung cancer care, and this may result in improved survival, guideline-based treatment, and quality of life for lung cancer patients. The assessment of guideline adherence and deviation could become a relevant and replicable standard criterion for cancer centers in Germany and globally. Single patient treatment may be optimized by systematic measurement and comparison of guideline and tumor board adherences as well as causes of their deviation and the applicability of these recommendations in clinical practice.

In the future, a special focus should be put on patients with SCLC as well as female patients.

## Informed Consent

Informed consent was obtained from all individual participants included in the study.

## Consent for publication

Consent for publication was obtained for every individual person’s data included in the study.

## Informed Consent

Informed consent was obtained from all individual participants included in the study.

## Consent for publication

Consent for publication was obtained for every individual person’s data included in the study.

## Supplementary Information

Below is the link to the electronic supplementary material.

Supplementary file1 (DOCX 977 KB)

## Data Availability

The datasets generated during and/or analyzed during the current study are available from the corresponding author on reasonable request.
