# Supplementary material for: Guideline adherence of tumor board recommendations in lung cancer and transfer into clinical practice
Source: J Cancer Res Clin Oncol. 2023 Jul 5;149(13):11679–88. doi: 10.1007/s00432-023-05025-1 (PMC10465379; doi:10.1007/s00432-023-05025-1)
Supplement: Supplementary file 1 — Supplementary file1 (DOCX 977 KB) [file 432_2023_5025_MOESM1_ESM.docx]

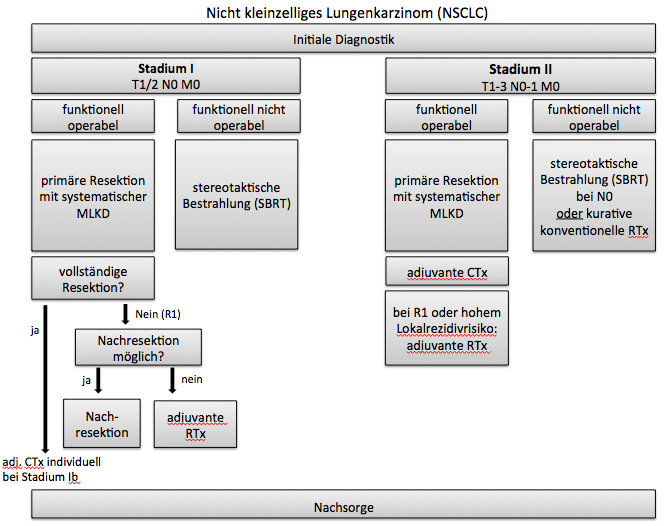
Notes: modified from SOP Lung cancer (Belka 2014) and (Goeckenjan et. al 2010).

**Figure 1 Supplement. Therapy recommendation in stage I and II NSCLC as recommended by the SOPs of the thoracic oncology center at the LMU Munich**

**Figure 2 Supplement. Therapy recommendation in stage IIIA NSCLC as recommended by the SOPs of the thoracic oncology center at the LMU Munich**


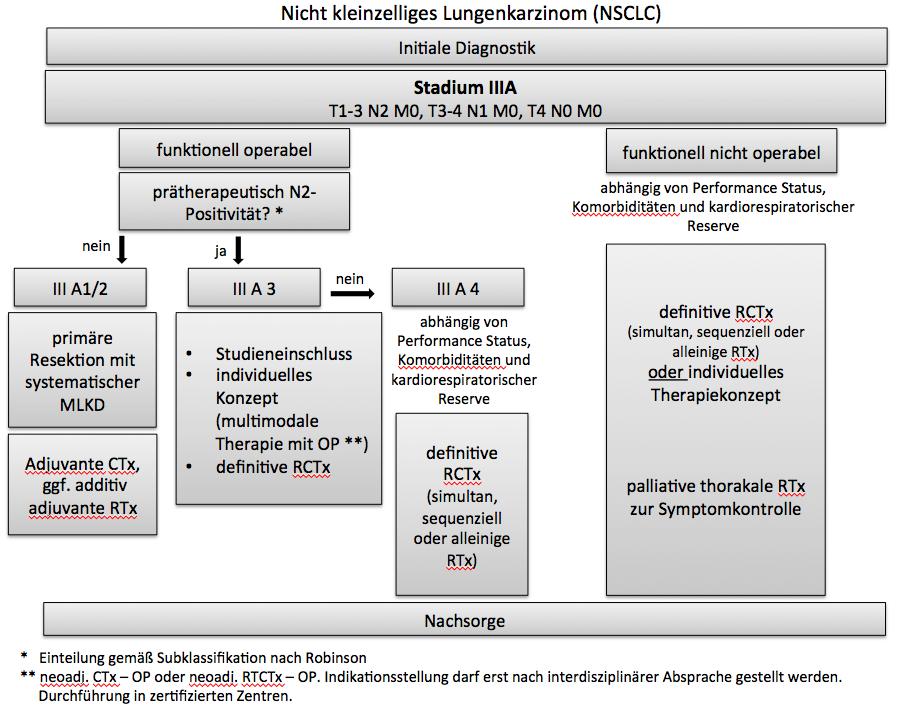


Notes: modified from SOP Lung cancer (Belka 2014) and (Goeckenjan et. al 2010).

CTx = chemotherapy, RTx = radiotherapy, MLKD = mediastinal lymph node dissection.

**Figure 3 Supplement. Therapy recommendation in stage IIIB NSCLC as recommended by the SOPs of the thoracic oncology center at the LMU Munich**


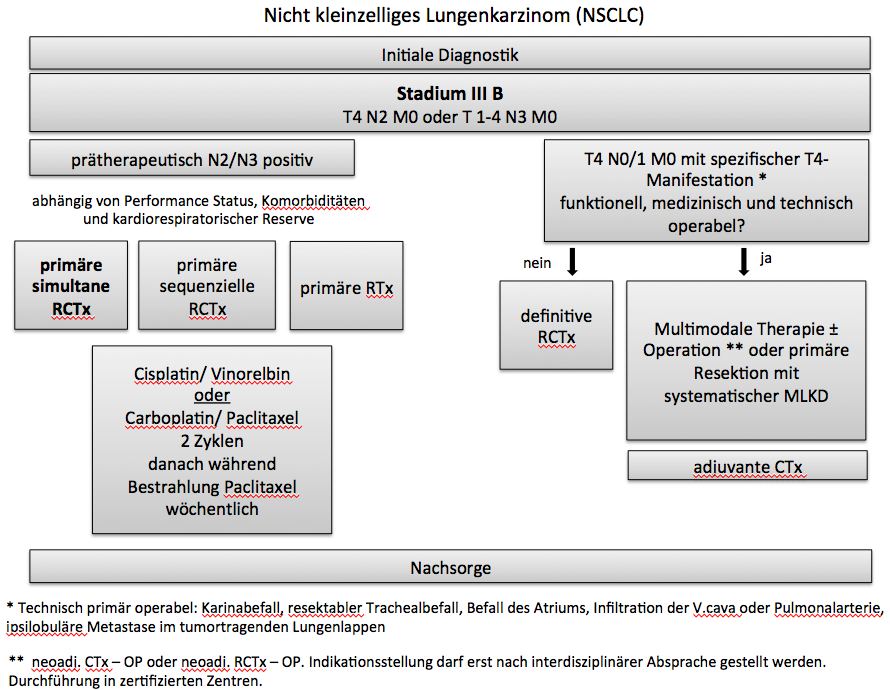


Notes: modified from SOP Lung cancer (Belka 2014) and (Goeckenjan et. al 2010).

CTx = chemotherapy, RTx = radiotherapy, MLKD = mediastinal lymph node dissection.

**Figure 4 Supplement. Therapy recommendation in stage IV NSCLC as recommended by the SOPs of the thoracic oncology center at the LMU Munich**


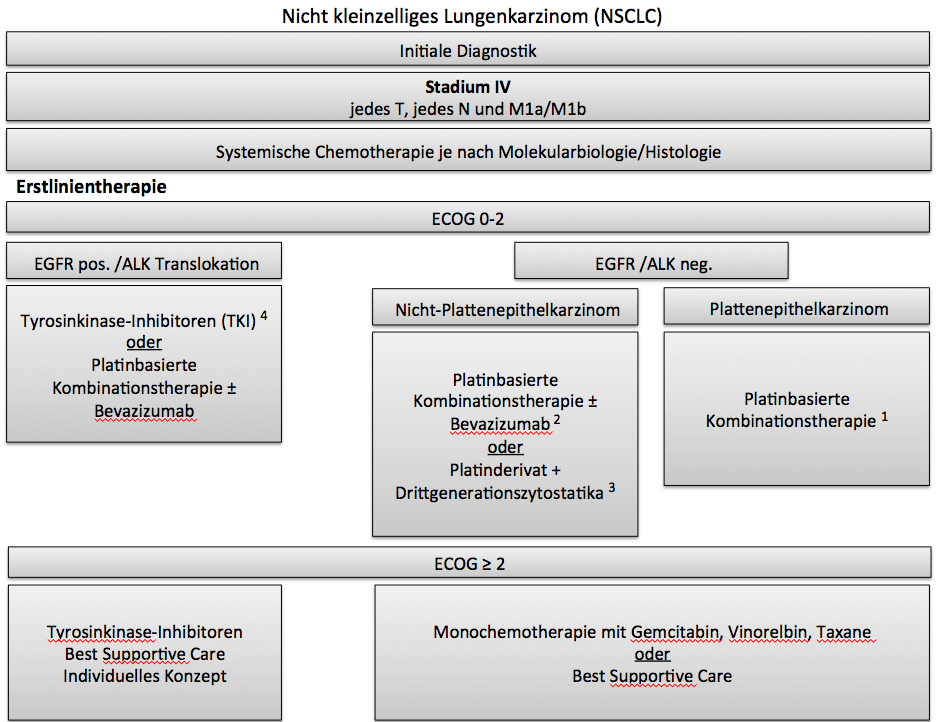

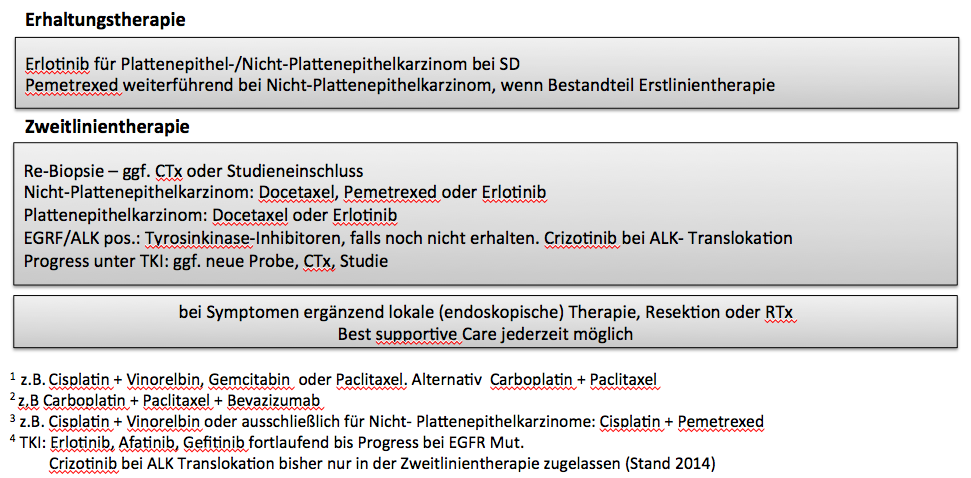


Notes: modified from SOP Lung cancer (Belka 2014) and (Goeckenjan et. al 2010).

**Figure 5 Supplement. Therapy recommendation in SCLC as recommended by the SOPs of the thoracic oncology center at the LMU Munich**


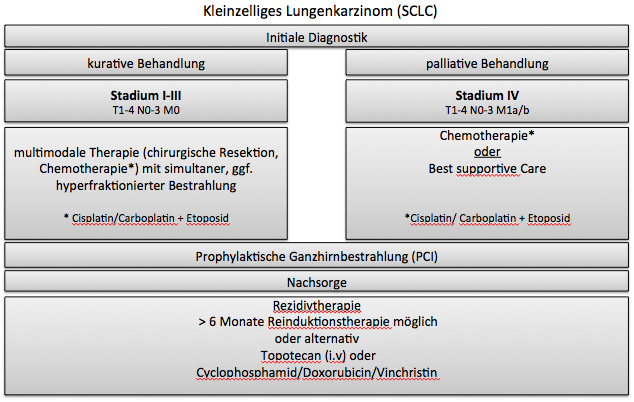


Notes: modified from SOP Lung cancer (Belka 2014) and (Goeckenjan et. al 2010).
